# Biological Sensing Using Vertical MoS_2_-Graphene Heterostructure-Based Field-Effect Transistor Biosensors

**DOI:** 10.3390/bios15060373

**Published:** 2025-06-10

**Authors:** Ying Chen, Nataly Vicente, Tung Pham, Ashok Mulchandani

**Affiliations:** 1 Department of Chemical and Environmental Engineering, University of California Riverside, Riverside, CA 92521, USA; nvice005@ucr.edu (N.V.); tpham052@ucr.edu (T.P.); 2 Center for Environmental Research and Technology (CE-CERT), University of California Riverside, Riverside, CA 92507, USA

**Keywords:** MoS_2_, graphene, heterostructures, FET, biosensor, biofunctionalization

## Abstract

Our study develops two configurations of MoS_2_ and graphene heterostructures—MoS_2_ on graphene (MG) and graphene on MoS_2_ (GM)—to investigate biomolecule sensing in field-effect transistor (FET) biosensors. Leveraging MoS_2_ and graphene’s distinctive properties, we employ specialized functionalization techniques for each configuration: graphene with MoS_2_ on top uses a silane-based method with triethoxysilylbutyraldehyde (TESBA), and MoS_2_ with graphene on top utilizes 1-pyrenebutyric acid N-hydroxysuccinimide ester (PBASE). Our research explores the application of MoS_2_–Graphene heterostructures in biosensors, emphasizing the roles of synthesis, fabrication, and material functionalization in optimizing sensor performance. Through our experimental investigations, we have observed that doping MoS_2_ and graphene leads to noticeable changes in the Raman spectrum and shifts in transfer curves. Techniques such as XPS, Raman, and AFM have successfully confirmed the biofunctionalization. Transfer curves were instrumental in characterizing the biosensing performance, revealing that GM configurations exhibit higher sensitivity and a lower limit of detection (LOD) compared to MG configurations. We demonstrate that GM heterostructures offer superior sensitivity and specificity in biosensing, highlighting their significant potential to advance biosensor technologies. This research contributes to the field by detailing the creation process of vertical MoS_2_–graphene heterostructures and evaluating their effectiveness in accurate biomolecule detection, advancing biosensing technology.

## 1. Introduction

Two-dimensional (2D) heterostructures are increasingly attracting attention for their unique ability to combine and enhance the individual properties of different 2D materials—such as graphene and molybdenum disulfide (MoS_2_)—through vertical stacking. This approach enables the creation of new material systems that combine distinct electronic, optical, and mechanical properties, allowing researchers to engineer new materials with tailored properties for advanced applications in electronics, optoelectronics, and biosensing [1,2,3]. Recent advances in the 2D sensing landscape have further broadened the applications and performance capabilities of such materials. For instance, miniaturized spectrometric systems based on Van der Waals heterostructures have demonstrated high-resolution spectral detection in compact formats, pushing the frontier of integrated optical sensing [4]. Additionally, the development of ultra-broadband multifunctional optoelectronics using engineered GaS–WSe_2_ heterostructures has enabled devices with enhanced photodetection across a wide spectral range, supporting multi-modal optoelectronics applications [5]. Meanwhile, progress in organic compound sensing has been exemplified by the functionalization of MXene-based surfaces, achieving high sensitivity and selectivity toward volatile organic compounds [6].

Within this growing field of 2D materials, graphene and MoS_2_ stand out for their complementary properties, making them ideal building blocks for heterostructure-based sensors. Graphene and MoS_2_ each contribute their unique advantages, setting the stage for groundbreaking applications. Graphene is renowned for its unparalleled electrical conductivity and robust mechanical properties, yet its zero bandgap is a limitation for semiconducting uses [7]. MoS_2_, with its significant bandgap and high on/off ratio, is well-suited for semiconducting applications but showed low conductivity and mechanical strength. Merging MoS_2_ and graphene into a heterostructure harnesses the best of both worlds—graphene’s efficient charge transfer and MoS_2_’s biosensing capabilities—effectively addressing their individual shortcomings with improved carrier mobility, sensitivity, and specificity for biomolecule detection and creating a synergistic platform for advanced biosensor development. The vertical stacking in 2D MoS_2_ and graphene heterostructures employs strong covalent bonds within planes and utilizes weaker Van der Waals forces between layers. This significantly broadens the potential applications of 2D materials by leveraging both the inherent properties of the individual layers and the novel features arising from their integration. Kim et al.’s study showcased the graphene/MoS_2_ heterojunction platform’s ability to significantly boost carrier mobility—up to ten times (~100 cm^2^/Vs) more than that of monolayer MoS_2_—while also maintaining a high on/off current ratio of about 10^8^ at room temperature [8]. This heterostructure’s advantages extend to adjustable Fermi levels via extensive back-gate bias, enabling the modulation of the Schottky barrier height, which, in turn, facilitates improved carrier mobility. Pham et al.’s research underscored the significant potential of graphene–MoS_2_ heterostructures in enhancing electronic devices, particularly for gas sensing applications [9]. The study specifically demonstrated advancements in transistor performance for detecting toluene, showcasing the heterostructures’ ability to improve the sensitivity and selectivity of sensors. This breakthrough paves the way for the development of more effective environmental monitoring and safety devices. Ignatova et al. explored the potential of MoS_2_–graphene heterostructures for label-free biosensing, targeting a widely used cancer medication [10]. Their comprehensive study revealed that lattice mismatches and variations in work function at the nanoscale significantly boost biosensor efficiency through three distinct optical detection methods. The research highlights the pivotal role of these heterostructures in enhancing sensitivity and specificity for biomolecule detection, signifying a major leap in biosensing technology.

Additionally, heterostructures offer more possibilities and choices for the biofunctionalization of biosensors, enhancing their adaptability and effectiveness in detecting a wide range of biological targets. Since there are two ways to build up the heterostructure biosensors, capping MoS_2_ on top of graphene functionalizes on MoS_2_, or capping graphene on top of MoS_2_ functionalizes on graphene. A variety of biofunctionalization techniques for MoS_2_-based biosensors have been explored, encompassing strategies like utilizing Van der Waals forces, leveraging hydrophobic interactions, taking advantage of electrostatic attractions, and covalent through diazonium salts doping and thiol coordination [11,12,13,14,15,16]. Similarly, grafted hydroxylated aryl group and covalent organic functional group approaches are widely applied for graphene functionalization [17]. Specially, non-covalence through π-systems essential for ensuring stability of functional nanomaterials and organic compounds is appealing as a production strategy owing to graphene’s unique natural stacking way [17,18,19,20].

Transistor-based sensors, particularly field-effect transistors (FETs), have garnered significant attention for their rapid response, high sensitivity, and compatibility with low-power, label-free detection platforms. Recent developments have demonstrated the versatility of FET architecture in various sensing applications. For instance, Shooshtari et al. reported an ammonia gas sensor utilizing multi-wall carbon nanofiber FETs with gate modulation, achieving a 55% improvement in sensor response and enhanced reversibility under optimal gate biases [21]. A study by Jin et al. introduced an ultrasensitive graphene FET biosensor utilizing a lithium niobate (LiNbO_3_) ferroelectric substrate. This innovative design achieved a detection limit as low as 1.7 femtomolar for microRNA-21, a recognized breast cancer marker, highlighting the efficacy of ferroelectric polarization in enhancing biosensor sensitivity [22]. Furthermore, comprehensive reviews by Smaani et al. and Wang et al. explored advancements in FET-based biosensor architecture and the application of organic electrochemical transistors (OECTs) for biomolecular detection, respectively [23,24]. These studies underscore the growing utility of transistor-based sensing platforms, supporting the approach adopted in this study.

In this paper, we explore the application of MoS_2_ and graphene vertical heterostructures in FET biosensors, focusing on the synthesis, functionalization, and characterization of these materials for biomolecule sensing. We explore how the arrangement of MoS_2_ and graphene layers affects their properties by alternating the stacking order of the two materials to obtain MoS_2_ on top of graphene (MG) and graphene on top of MoS_2_ (GM) configurations, examining their electronic interactions, charge transfer dynamics, and the implications for carrier mobility and biosensor performance. Functionalization techniques are tailored to each material’s unique properties with two-step processes: MoS_2_ involved silane-based triethoxsilylbutyraldehyde (TESBA) deposition on the surface, followed by mobilization of antibody with the aldehyde of TESBA; while graphene was modified with self-assembled 1-pyrenebutyric acid N-hydroxysuccinimide ester (PBASE), followed by a reaction between the aldehyde of PBASE with antibody. By systematically studying the influence of material characteristics and device architecture on biosensing performance, the optimal configuration depends on the specific application’s requirements, such as desired electronic characteristics, carrier mobility, or sensitivity. This work sheds light on the mechanisms underpinning biomolecule detection and offers insights into optimizing heterostructure-based FET biosensors for clinical and environmental applications.

## 2. Materials and Methods

### 2.1. Material Synthesis

MoS_2_ was synthesized on a 300 nm thick SiO_2_ substrate utilizing MoO_3_ and sulfur as precursors in a chemical vapor deposition (CVD) chamber. The process was conducted under an argon gas atmosphere to ensure controlled growth conditions. Graphene synthesis was carried out separately in a distinct CVD chamber. This process involved the use of a polycrystalline copper foil as the growth substrate, with hydrogen (10 sccm) and argon (300 sccm) gases creating the necessary chemical environment. A low concentration of methane (90 ppm) was introduced as the carbon source for graphene growth.

The synthesized graphene protected by a polymer layer was gently lifted off in 0.2 M ammonium persulfate solution. After a thorough rinse with distilled water to eliminate contaminants, the graphene was carefully transferred onto a SiO_2_ substrate for further experimentation and analysis.

### 2.2. Fabrication of FET Devices

The fabrication of FET devices commenced with the precise patterning of source and drain electrodes on Si/SiO_2_ substrates using e-beam lithography. It was followed by the vapor deposition of a 5/50 nm Cr/Au layer onto the substrates, establishing the required electrical contacts for the FETs.

MoS_2_/Graphene (MG) heterostructure fabrication: The assembly of MoS_2_ layers onto the metal electrodes was achieved using a polystyrene (PS) transfer film. After the MoS_2_ layer assembly, the device underwent vacuum drying and was subsequently immersed in toluene to dissolve and remove the PS layer. To ensure contact between MoS_2_ and the electrodes, the device was annealed at 200 °C for 2 h in a hydrogen (10 sccm) and argon (300 sccm) atmosphere. The graphene layer, carried by a polymethyl methacrylate (PMMA) film, was then accurately aligned, and placed atop the MoS_2_. This graphene layer application followed a similar procedure of vacuum drying and acetone immersion. A final annealing step at 200 °C for 2 h under hydrogen and argon gases was essential to ensure the successful integration of the MoS_2_ and graphene layers.

**Figure 1:**
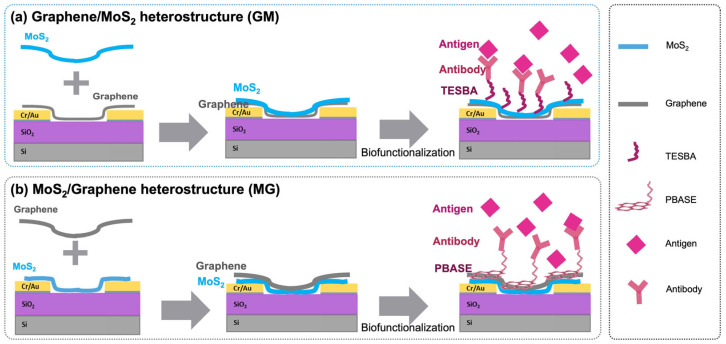
Schematic illustration of the FET devices’ fabrication, antibody immobilization, and antigen detection of graphene and MoS_2_ heterostructure-based biosensors.

Graphene/MoS_2_ (GM) heterostructure fabrication: The fabrication of the GM heterostructure paralleled that of the MG heterostructure, with a key distinction being the sequence of material transfer as illustrated in Figure 1. Initially, the graphene layer was transferred to the substrate using a PMMA film, and this step was followed by the transfer of the MoS_2_ layer using a PS film. Both transfer steps required careful alignment, application of the polymer films, vacuum drying, acetone and toluene immersion to remove the polymers, and annealing to achieve a cohesive and stable heterostructure. The fabrication process did not involve photolithographic patterning of either graphene or MoS_2_; instead, both materials were manually aligned and transferred to define the device geometry.

### 2.3. Functionalization

MG FET Device Functionalization: The functionalization of the MG FET device’s graphene surface involved several carefully executed steps. Initially, the surface was treated with 20 μL of a 10 mM solution of PBASE (Sigma-Aldrich, St. Louis, MO, USA) in dimethylformamide (DMF) for 2 h. PBASE molecules were non-covalently bonded to the graphene surface through π–π stacking interactions, leveraging the complementary aromatic structures of graphene and pyrene in PBASE [25]. Following this, the device underwent sequential rinses with DMF, methanol, and deionized water to eliminate unbound PBASE. Subsequently, the device’s channel was incubated with 20 μL of a 50 μg/mL anti-human immunoglobulin G (HIgG, Sigma-Aldrich, St. Louis, MO, USA) antibody solution in 0.1× phosphate-buffered saline (PBS) at 4 °C for 12 h. This step enabled the covalent attachment of antibodies to the surface-bound PBASE molecules via amide bond formation with the primary amine groups on the antibodies. The device was then rinsed with a 10 mM phosphate buffer (PB) at pH 7.4. The successful attachment of PBASE to graphene and antibodies to PBASE was confirmed using Raman spectroscopy and atomic force microscopy (AFM). Finally, 20 μL of 0.1 M ethanolamine in PB was applied to each sensing site and incubated at room temperature for 30 min, followed by a PB rinse.

GM FET Device Functionalization: The GM FET devices underwent a different functionalization process. Devices were submerged in a 2% (*v*/*v*) TESBA (Gelest Inc., Morrisville, PA, USA) solution in toluene in sealed containers, reacting for 2 h to allow for silane deposition [14]. Post-reaction, the devices were rinsed with ethanol and deionized water and subsequently dried. A back-dried step at 120 °C for 20 min was then performed [26]. Antibody immobilization was carried out similarly to the MG FET devices, involving the immobilization of anti-HIgG antibodies in PBS to the channel, followed by incubation and rinsing with PB. The successful coupling of TESBA to MoS_2_ and antibodies to TESBA was confirmed by X-ray Photoelectron Spectroscopy (XPS) and AFM. Ethanolamine treatment was performed as with the MG FET devices, involving incubation and subsequent rinsing. The detection molecules were applied globally to the exposed surface of the vertical heterostructure devices for both configurations.

### 2.4. Optical and Electrochemical Characterization

Optical microscopy images were captured with a Nikon L150 Optical Microscope (Nikon Corporation, Tokyo, Japan). Raman spectroscopy was carried out using a Horiba LabRam system with a 532 nm green laser with a minimal power of 5 mW as source, enabling the identification of molecular vibrations and chemical composition. Atomic force microscopy (AFM) images were obtained using a Horiba LabRam/Aist-NT AFM system (Horiba LabRam HR, Kyoto, Japan). XPS measurements were conducted using a Kratos AXIS ULTRADLD XPS system (Kratos Analytical, Manchester, UK) with an Al Kα X-ray source and a 165 mm mean radius electron energy hemispherical analyzer. Vacuum pressure was meticulously maintained below 3 × 10^−9^ torr during the measurements. The Fourier transform infrared spectroscopy (FT-IR) spectra were obtained using a Nicolet 6700 FT-IR spectrometer (Thermo Scientific, Temecula, CA, USA).

FET transfer characteristic curves were acquired using a Keithley 2636 source meter from Tektronics (Beaverton, OR, USA). In this setup, a constant drain–source voltage (V_D_) of 1 V was maintained while the back-gate voltage (V_G_) was systematically swept from 0 V to 120 V. A 5 nm/50 nm Cr/Au layer was deposited on the backside of the silicon substrate to serve as the back-gate electrode. For resistance measurements, resistance was calculated from the linear region of the current–voltage curves, which were meticulously recorded across a range from −0.1 V to 0.1 V. To accurately evaluate the real-time current responses and specificity of our FET biosensors, resistance-against-time (R-t) measurements were employed. These measurements were instrumental in assessing the biosensors’ ability to distinguish between molecules similar to the targeted analyte. For specificity assessment of the sensing platform, bovine serum albumin (BSA, Sigma-Aldrich, St. Louis, MO, USA) and human serum albumin (HSA, Sigma-Aldrich, St. Louis, MO, USA) were selected as control proteins. A commercial Ag/AgCl reference electrode was used for liquid-gating during biosensing measurements. Additionally, the R-t measurements played a key role in monitoring the biosensors’ response to various concentrations of HIgG antigen. For real-time detection measurements, a constant bias V_D_ = 1 V was applied without gate voltage. To define the sensing region and contain the analyte solution, a custom 30 μL PMMA well was fabricated and mounted onto the device. The well featured a rounded rectangular shape with internal dimensions of approximately 6 mm × 3 mm and a depth of 1.7 mm. The design and application of similar PMMA-based fluidic structures for biosensing measurements have been demonstrated in our previous work [27]. Before the introduction of the samples, each FET biosensor underwent a stabilization period of 10–15 min to establish a baseline for current response. Following this stabilization, 200 µL of the sample, prepared in 10 mM PB, was sequentially added to the PMMA cell at 6 to 7 min intervals. This methodical addition of samples was critical for eliciting stable and reliable current responses from the biosensors, thereby ensuring the accuracy and reproducibility of our measurements. All concentration-dependent measurements were performed on the same individual device to eliminate the influence of device-to-device variability.

## 3. Results and Discussion

### 3.1. Materials Characterizations

**Figure 2:**
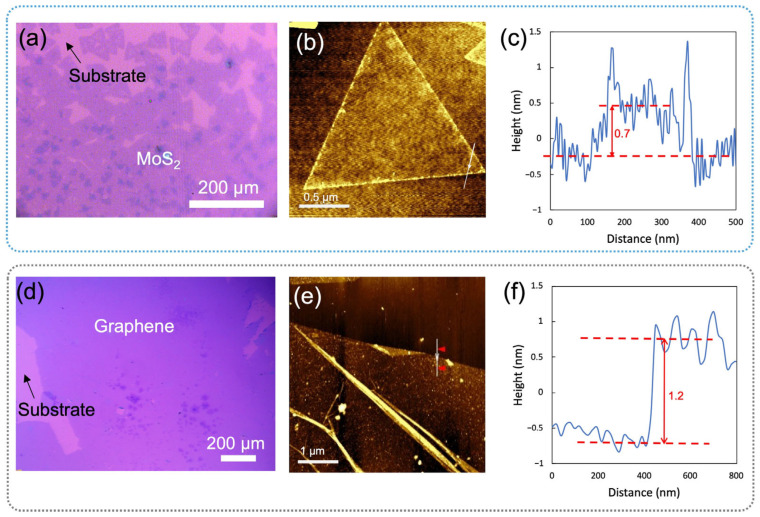
Optical images and AFM of MoS_2_ and graphene: (**a**) a CVD-grown MoS_2_ on a Si/SiO_2_ substrate, (**b**) an AFM image of a MoS_2_ flake with (**c**) its height profile, and (**d**) graphene synthesized via CVD on a Si/SiO_2_ substrate, along with (**e**) its AFM image and (**f**) the corresponding height profile.

**Figure 3:**
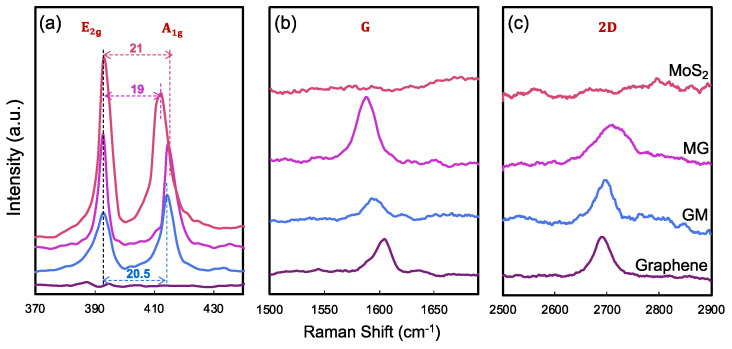
Raman spectra of MoS_2_, graphene, and heterostructures. (**a**) MoS_2_ spectra showing E_2g_ and A_1g_ modes. (**b**) G band region and (**c**) 2D band region of graphene.

Both bare MoS_2_ and graphene were synthesized using the CVD method and characterized by optical images and AFM (Figure 2). The MoS_2_ was grown directly on Si/SiO_2_ substrate in continuous film and mainly comprises a single layer. The small MoS_2_ flake was characterized by AFM with a thickness of about 0.7 nm (Figure 2c). In Figure 3a, the Raman spectra of CVD-MoS_2_ highlight two distinct peaks: E_2g_ at 393 cm^−1^ and A_1g_ at 415 cm^−1^, representing the in-plane and out-of-plane vibrational modes. The notable separation of 19 cm^−1^ between these peaks indicates the monolayer structure of MoS_2_ [28]. The uniform color contrast in Figure 2d indicates that the graphene is a continuous film. The height between the substrate and the graphene surface indicates the thickness of the graphene was 1.2 nm. Raman spectra in Figure 3b, c of our CVD graphene show a strong G peak at 1606 cm^−1^ and 2D peak at 2670 cm^−1^. Raman spectra also denoted there were no defects introduced during both the growth phase and the subsequent transfer of the materials.

Following the synthesis of each material separately, the heterostructures were subsequently constructed through physical stacking by capping graphene with MoS_2_ (in which MoS_2_ is underneath graphene, shortened to MoS_2_/Graphene or MG as illustrated in Figure 1a), and capping MoS_2_ with graphene (in which graphene is underneath MoS_2_, shortened to Graphene/MoS_2_ or GM as illustrated in Figure 1b). Raman spectroscopy confirmed that the intrinsic properties of both materials remained intact post-transfer, ensuring their quality and compatibility within the heterostructure. The blueshift of A_1g_ mode (Figure 3a) in both MG and GM heterostructures indicates the existence of a Van der Waals interaction at the interface [29,30]. Figure 3b compares the G band of graphene in all cases. The G peak position shows a much larger shift of the GM compared with the case of MG. The possible reason is that graphene becomes more sensitive when it is in contact with different bottom and top dielectric environments [29,30]. Since the measured Raman G peak position shifts Δ*Ω*_G_ linearly with Fermi energy change Δ*E*_F_, Δ*Ω*_G_ = |Δ*E*_F_| × 42 cm^−1^ eV^−1^ [30,31]. The large shift of the G band is the result of the stacking order and intimate interface. The stacking of MoS_2_ on graphene leads to a much larger shift of the graphene G band, compared with the case of graphene on MoS_2_. For MG, G peaks downshift for 16 cm^−1^, corresponding to a Fermi level shift of 0.38 eV in graphene due to contact with MoS_2_, i.e., electron transfer from MoS_2_ to graphene lifts the graphene Fermi level by 0.38 eV; while for GM, G peaks downshift for 8 cm^−1^, reflecting a Fermi level shift of 0.19 eV in graphene due to contact with MoS_2_, i.e., electron transfer from MoS_2_ to graphene lifts the graphene Fermi level by 0.19 eV. The possible reason is that graphene becomes more sensitive when it is in contact with different bottom and/or top dielectric environments [29,30]. The positions of the 2D peaks were also a function of doping, as shown in Figure 3c, where both heterostructures’ 2D peak show blueshift, which means interaction within both heterostructures results in the doping of the graphene layer [9,32]. The 2D peak upshifts by 55 cm^−1^ for MG and the 2D peak upshifts by 45 cm^−1^ for GM, and the doping in MG is stronger than the doping in the GM structure.

**Figure 4:**
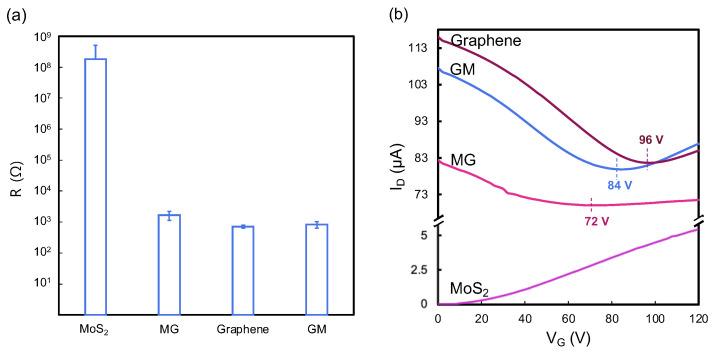
(**a**) Resistance results and (**b**) representative transfer curve of MoS_2_, graphene, and MG and GM heterostructures.

To enhance our comprehension of the electrical properties of heterostructures, we conducted comparative analyses of their resistances. In Figure 4a, given that graphene is known for its high conductivity and low resistance, ~700 Ω, in contrast to MoS_2_, which exhibits a high resistance of ~170 MΩ, the assembly of these materials into heterostructures provides insightful observations. The MG heterostructure demonstrated a resistance of ~1700 Ω, whereas the GM heterostructure showed a resistance ~800 Ω. This suggests the electrical performance of the heterostructures remains predominantly governed by the properties of graphene, indicating that the simple physical stacking method does not significantly alter the conductive dominance of graphene within these composite structures.

To further investigate the effects of doping on electronic performance, this study assessed the electronic characteristics of backgated FETs made from these materials. The FET transfer curves were plotted in Figure 4b. The high p-doping graphene transfer curve shows a charge neutrality point of gate voltage (V_CNP_) at 96 V. The significant p-doping of graphene is frequently seen because of the photolithography process used for device patterning which typically incorporates steps of exposure to plasma and immersion in metal etching agents [9]. The presence of ambient adsorbents like water vapor and oxygen can further enhance the p-doping phenomenon. The noticeable difference of slopes in the right and left arms of the transfer curve indicates the as-fabricated CVD graphene dominated by hole carriers. The MoS_2_ FET curve shows the typical n-type performance of CVD-grown MoS_2_ at room temperature.

Both heterostructure FETs show ambipolar transfer characteristics and exhibit a shift in V_CNP_ compared with graphene. The V_CNP_ shifts to 84 V for GM, indicating that graphene becomes less p-doped but more n-doped by MoS_2_. This phenomenon, where graphene capped with MoS_2_ exhibits a shift from p-type to more n-type characteristics, is attributed to the migration of electrons from MoS_2_ to graphene. Under these conditions, the normally p-doped multilayer graphene serves as a recipient for electrons originating from the n-doped MoS_2_. A similar report on the electronic properties of MoS_2_ and graphene heterostructures was illustrated in our previous work [9]. On the other hand, the V_CNP_ is found at 72 V for MG which shows more n-doped effect by the MoS_2_ compared with graphene and the GM heterostructure. This discrepancy highlights the impact of physical stacking techniques on the interactions and charge transfer within the heterostructure. Such influences are critical in altering carrier mobility, thereby directly affecting the behavior of charge carriers within the heterostructure.

### 3.2. BioFunctionalization

One advantage of using heterostructures in biosensors lies in the capacity to leverage the unique properties of both constituent materials, enabling specialized functionalization techniques to be tailored for each layer. By capitalizing on the distinct attributes of MoS_2_ and graphene, we implement specific functionalization strategies that are designed to complement the unique characteristics of each material in various configurations. For graphene with MoS_2_ on top, we utilize a silane-based method that involves TESBA, while for MoS_2_ with graphene on top, we utilize a PBASE-based approach. This deliberate choice ensures that the functionalization process is perfectly attuned to the inherent properties of the material on the upper layer of each heterostructure. Consequently, we compare the two approaches for the biofunctionalization of graphene and MoS_2_ through a two-step biofunctionalization process, characterized by XPS, Raman spectroscopy, and AFM characterizations.

**Figure 5:**
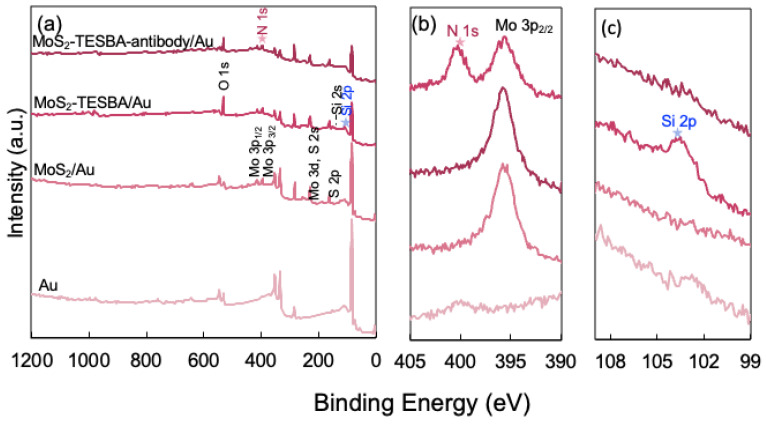
(**a**) XPS survey spectra of MoS_2_ at different stages of functionalization spotted on a gold substrate. (**b**) High-resolution XPS spectra of N 1s and Mo 3p peaks. (**c**) High-resolution XPS spectra of Si 2p peaks.

To determine the chemical composition of MoS_2_ modified with TESBA, XPS spectral analysis was conducted on gold substrate. The wide-scan spectra of both MoS_2_/Au, MoS_2_–TESBA/Au, and MoS_2_–TESBA–antibody/Au as shown in Figure 5a, reveal the presence of Mo, S, C, and O elements. These elements are observed in the MoS_2_/Au, MoS_2_–TESBA/Au, and MoS_2_–TESBA–antibody/Au samples. Notably, the Si element is detected in the MoS_2_–TESBA sample (as detailed in Figure 5c), confirming the successful incorporation of TESBA onto the MoS_2_ surface. The increase in the O 1s signal further indicates that TESBA molecules are crosslinked, as evidenced by the presence of Si–O–Si bonding on the surface. Since TESBA is the sole source of both silicon and oxygen, these signals serve as reliable indicators of its presence and the degree of crosslinking density [14]. Furthermore, a significant increase in the N 1s peak intensity (Figure 5b) is observed on the MoS_2_–TESBA–Antibody/Au surface, which is attributed to the immobilization of antibodies. This enhancement in the N signal reflects the abundance of nitrogen-containing peptide bonds in the antibody molecules, thereby confirming successful biofunctionalization of the surface [33]. The peak observed at 401 eV could be an overlap between N1s and Mo 3p.

**Figure 6:**
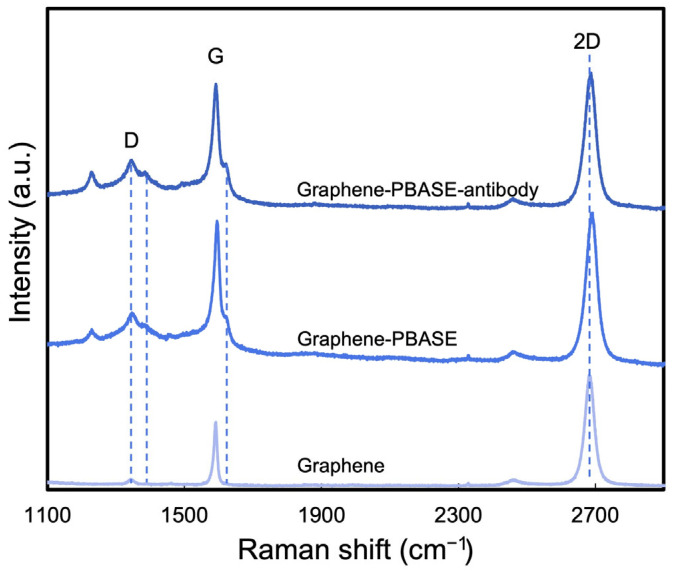
Raman spectra of bare graphene, PBASE-functionalized graphene, and antibody-immobilized graphene.

Raman spectroscopy was applied to confirm the presence of PBASE on the surface of the FET biosensor. Figure 6 displays the averaged Raman spectra for bare graphene, PBASE-functionalized graphene, and antibody-immobilized graphene, derived from multiple measurements across each sample to thoroughly assess the impact of PBASE functionalization on graphene quality. Before functionalization, the D peak at 1350 cm^−1^ was observed, typically associated with graphene’s structural defects. After PBASE functionalization, this D peak intensified and broadened, indicating increased structural irregularities. Furthermore, PBASE-specific intrinsic signals emerged, notably a peak at 1626 cm^−1^ corresponding to pyrene group resonance and a peak at 1401 cm^−1^, reflecting disorder from orbital hybridization with the graphene plane [34]. A notable shift (~7 cm^−1^) in the 2D peak frequency of the graphene films to a higher value (as illustrated in Figure 6) suggests hole doping by PBASE [19,25]. While the antibody immobilization’s doping effect on both the G band and the 2D peak was less pronounced than that of PBASE, it nonetheless led to enhanced signals at 1357 cm^−1^ and 1401 cm^−1^. Additionally, a subtle shift (~3 cm^−1^) towards a lower frequency in the 2D peak was observed following antibody immobilization, further indicating the interaction between the antibody and the PBASE-functionalized graphene surface.

**Figure 7:**
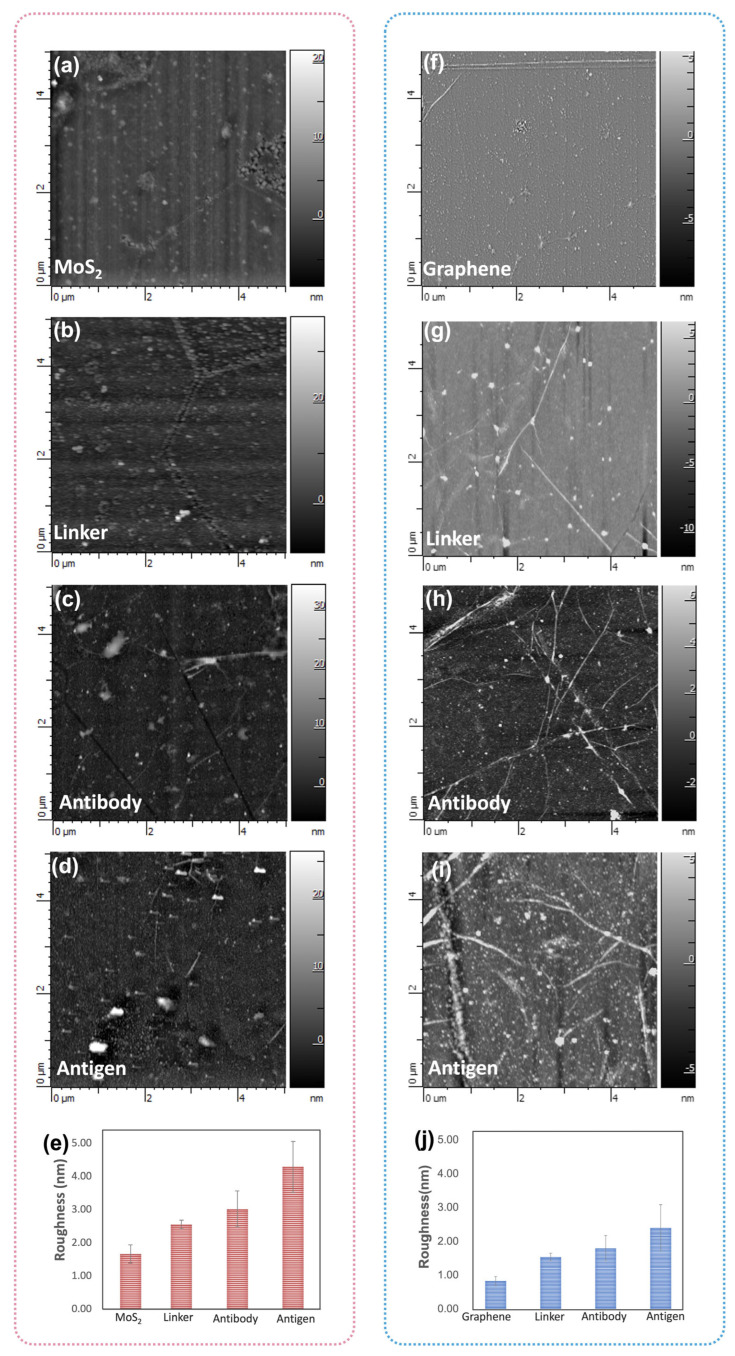
The AFM image of (**a**) bare MoS_2_, (**b**) TESBA conjunctions, (**c**) after antibody immobilization, and (**d**) after antigen attachment of the MoS_2_ surface. (**e**) The RMS roughness changes of the MoS_2_ surface functionalization process. The image of (**f**) bare graphene, (**g**) PBASE conjunctions, (**h**) after antibody immobilization, and (**i**) after antigen attachment of the graphene surface. (**j**) The RMS roughness changes of the graphene surface functionalization process. AFM images were obtained within a 5 μm × 5 μm area.

AFM is employed to examine the alterations in surface topography resulting from the biofunctionalization process. Figure 7a–d present the AFM images of the bare MoS_2_, TESBA conjunctions, after antibody immobilization, and after antigen attachment, respectively. We observe small clusters of thicker materials attributed to non-uniform MoS_2_ growth on the edge, which contributes to more active sites for functionalization. Bare MoS_2_ exhibits a root mean square (RMS) roughness less than 1.7 nm. After the TESBA conjunctions, the surface roughness increases to 2.5 nm. Subsequently, the surface roughness was noted to elevate to 3 nm following antibody immobilization, and a further increase of 4.1 nm was observed upon the attachment of antigens. Figure 7f–j shows the changes of graphene biofunctionalization which resulted in the morphology of the surface after the linker modification, the immobilization of the antibody, and the capture with antigen. Ripples and wrinkles observed in large-area graphene (as shown in Figure 7f–i) are a result of the transfer process. Additionally, the presence of small clusters of thicker materials is identified as residues which were not eliminated during the cleaning and transferring procedures of the graphene. The surface roughness changes during biofunctionalization of graphene are nearly analogous to MoS_2_, which shows a progressive increase after antibody immobilization and antigen detection (Figure 7j) [35,36].

### 3.3. Biosensing Performance

**Figure 8:**
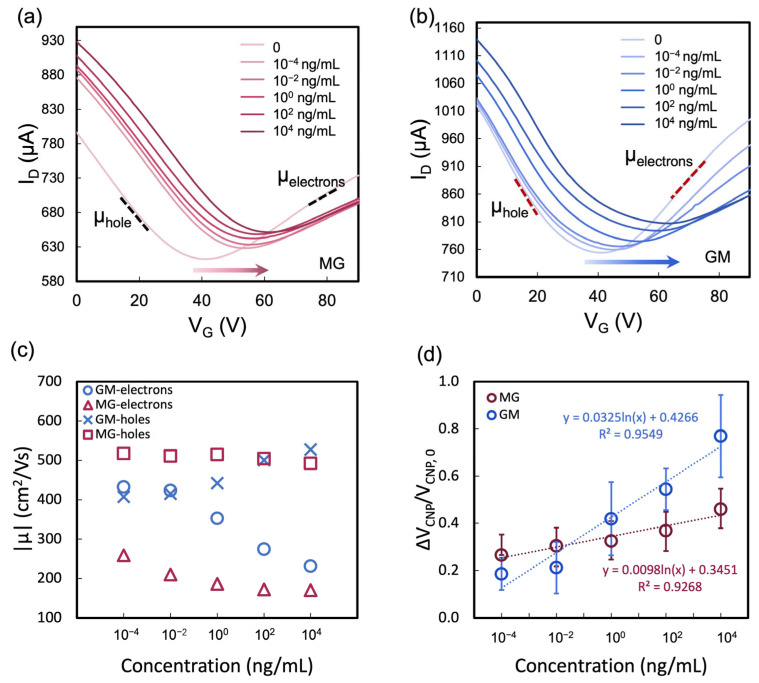
(**a**) Representative transfer curves for various concentrations of the HIgG antigen using (**a**) an MG FET biosensor and (**b**) a GM FET biosensor. (**c**) Calculated carrier mobility from the linear regions of the transfer curves in (**a**,**b**). (**d**) Concentration-dependent calibration curves for the HIgG antigen, demonstrating the shifts in the charge neutrality point of gate voltage (ΔV_CNP_) for both MG FET (red line) and GM FET (blue line) biosensors. Data and error bars represent the average ± standard deviation of responses from 5 individual sensors.

Transfer curves of the two heterostructures biosensors were obtained to detect varying concentrations of HIgG analyte. The sensing properties were assessed through the shift in the V_CNP_, which is defined as ΔV_CNP_ = V_CNP_ − V_CNP0_, where V_CNP0_ and V_CNP_ are the charge neutrality point before and after the addition of the target. As with most ambient-condition electrical measurements in 2D materials, the influence of surface adsorbates cannot be entirely excluded, though relative trends remain consistent. Upon introduction of a low HIgG concentration (10^−4^ ng/mL), a discernible rightward shift was observed for both MG (Figure 8a) and GM (Figure 8b) heterostructure biosensors coupled with a decrease in the overall current level. At higher HIgG concentrations, the transfer curves exhibited further rightward shifts, and the V_CNP_ values increased accordingly. These shifts and the current changes suggest that HIgG binding to the functionalized heterostructure surface results in changes to the local charge environment. The interaction of HIgG with the biosensor’s surface receptors leads to an alteration in the electrostatic environment of the heterostructure surface, impacting charge carrier distribution. These changes are sensitively detected by the FET, which reflects them as variations in carrier mobility. Specifically, the binding event can lead to a change in the carrier mobility, which can be calculated from the linear region of the transfer curves for each analyte concentration and summarized in Figure 8c. The mobility (*μ*) of charge carriers in a FET was calculated using the following equation [37]:

$$\mu =\frac{g_{m}L}{WC_{ox}V_{D}}$$

where *g_m_* is the transconductance, which is the slope of the transfer curves in the linear region. *L* and *W* are the length and width of the channel, which are both specified as 10 μm. *C_ox_* is the capacitance per unit area of the gate oxide. For a gate oxide composed of 300 nm thick SiO_2_, *C_ox_* has been calculated to be 1.15 × 10^−8^ F/cm^2^ [38]. In comparing the sensing performances of the MG and GM heterostructure biosensors when detecting HIgG, distinct behaviors were observed in response to increasing analyte concentrations. For the MG heterostructure, the mobility of holes remained largely unchanged, while there was a slight decrease in electron mobility. In contrast, the GM heterostructure exhibited an increase in hole mobility and a decrease in electron mobility as the concentration of HIgG analyte increased. This variance suggests that the electrostatic environment created by the analyte–receptor complex has a more pronounced impact on the GM heterostructure, significantly affecting the distribution of charge carriers. This difference in response highlights the unique electronic interactions occurring within each heterostructure upon HIgG binding. In addition, the distinct linker chemistries used for functionalization—TESBA for MoS_2_ in GM and PBASE for graphene in MG—may influence the local electrostatic environment at the sensing interface and potentially contribute to the observed variation in response. The calibration curves in Figure 8d, which were derived from the shifts in the V_CNP_, provide further insights into the biosensors’ performance and offer a comprehensive overview of the biosensors’ responses across a range of HIgG concentrations. A linear relationship is observed between the shift in ΔV_CNP_ and the concentration HIgG, which fits to the linear equation for both heterostructures, y = 0.0325lnx + 0.4266 for GM heterostructure and y = 0.0098lnx + 0.3451 for MG heterostructure FET biosensors. The limit of detection (LOD) was determined following the same method described in our previous work [27,39]. The results showed that the GM biosensor has higher sensitivity (0.0325 vs. 0.0098 per ln[HIgG, ng/mL]) and lower LOD (1.24 × 10^−5^ ng/mL vs. 2.64 × 10^−5^ ng/mL) compared to the MG biosensor. Both heterostructures’ FET biosensors exhibit comparable LODs to existing MoS_2_ FET biosensors, as summarized in Table 1. Furthermore, they have demonstrated higher LODs than simple graphene FET biosensors for antibody detection.

Beyond the works included in Table 1, this study also offers notable advancements compared to prior studies involving MoS_2_–graphene heterostructures. Kim et al. [8] reported high-mobility junction FETs based on graphene/MoS_2_ interfaces, focusing on fundamental electronic transport characteristics but without exploring biosensing applications. Ignatova et al. [10] investigated charge redistribution in vertical 2D heterostructures using multidimensional imaging, providing mechanistic insights into biosensing interactions, but without evaluating electrical performance or comparing device architectures. In contrast, our study utilizes a FET platform for real-time, label-free biosensing and directly compares two vertical stacking configurations within a consistent experimental framework. This comparative analysis reveals how stacking order influences charge transport, sensitivity, and detection dynamics, offering practical design insight for future 2D-material–based biosensors.

**Figure 9:**
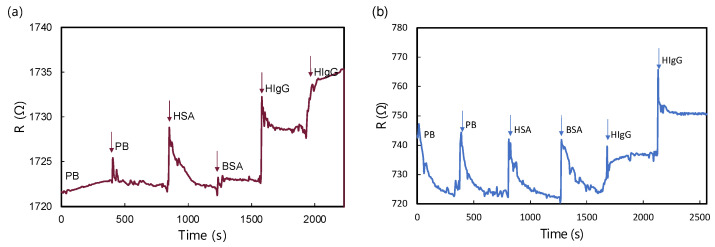
R-t measurement of (**a**) MG biosensor and (**b**) GM biosensor upon the addition of different biomolecular solutions. All analyte solutions prepared in 10 mM PB consisted of 10^2^ ng/mL of HSA, 10^2^ ng/mL of BSA, 10^2^ ng/mL, and 10^4^ ng/mL of HIgG.

Figure 9 offers an insightful analysis of the real-time resistance responses exhibited by MG and GM biosensors when exposed to various biomolecular solutions. Both biosensors were tested against solutions of HSA, BSA, and HIgG. For both MG and GM heterostructure biosensors, no obvious change in resistance was observed upon the addition of bare PB and non-target biomolecules of HSA and BSA in high concentrations. Conversely, a noticeable change in resistance was observed upon the addition of HIgG solutions, indicating a strong interaction between the biosensor surface and the HIgG molecules. The response to HSA and BSA, on the other hand, was relatively muted, suggesting a high degree of specificity of the MG biosensor towards HIgG. This specificity is crucial for applications where accurate detection of HIgG is essential. Furthermore, the analysis of resistance changes at different concentrations of HIgG (10^2^ ng/mL and 10^4^ ng/mL) provides valuable insights into the biosensors’ sensitivity. Both MG and GM biosensors exhibited a concentration-dependent response, with higher concentrations of HIgG leading to more significant changes in resistance. Additionally, both configurations showed rapid responses, with response times between 70 and 100 s. This concentration-dependent behavior is indicative of the potential of these biosensors for quantitative analysis that requires high specificity and sensitivity in clinical and diagnostic applications.

## 4. Conclusions

In conclusion, we have presented an experimental study on biomolecule sensing using vertical MoS_2_–graphene heterostructure-based FET biosensors, highlighting the synthesis and fabrication processes of MoS_2_ and graphene via CVD, and the subsequent assembly into MG and GM heterostructures. We employed specialized functionalization techniques for each configuration tailored to each topping material’s unique properties: graphene with MoS_2_ on top uses a silane-based method with TESBA, and MoS_2_ with graphene on top utilizes PBASE. Biofunctionalization techniques for both heterostructures are detailed, leading to specific biomolecular detection. Surface characterizations and FET device performance are thoroughly investigated. Our study demonstrates that GM heterostructures exhibit higher sensitivity and lower LOD for HIgG antigen compared to MG configurations, with GM showing a sensitivity of 0.0325 vs. 0.0098 per ln[HIgG, ng/mL] and a LOD of 1.24 × 10^−5^ ng/mL vs. 2.64 × 10^−5^ ng/mL for MG. This suggests the GM configuration’s superior performance in biosensing due to its enhanced charge carrier distribution when interacting with biomolecules. This study showcases the potential of these heterostructures in biosensing applications, with distinct advantages in carrier mobility and limit of detection highlighted through comprehensive data analysis.

In addition to demonstrating high sensitivity, specificity, and rapid response times, the proposed vertical MoS_2_ and graphene heterostructure biosensors offer promising potential for integration into practical diagnostic platforms. The compact device design and low sample volume requirement make the system highly compatible with microfluidic integration, enabling automated sample handling, improved fluid control, and multiplexed detection—key features for portable and user-friendly point-of-care diagnostic tools. Moreover, the use of inherently flexible 2D materials renders the sensor architecture well-suited for fabrication on flexible substrates, opening opportunities for wearable and conformable sensing formats. These formats are particularly attractive for continuous and personalized health monitoring. In addition, the biosensor platform is adaptable; the recognition elements and target molecules can be modified to detect a broad range of biomolecular analytes. Overall, this work provides valuable insights for future efforts aimed at enhancing the versatility, scalability, and real-world applicability of graphene–MoS_2_-based biosensors.

## Figures and Tables

**Table 1:** Summary and comparison of MoS_2_, graphene, and graphene and MoS_2_ heterostructure FET biosensors for antibody detections.

Materials	Antibody Type	Detection Range	LOD or Detection Limit	Reference
MoS_2_	prostate-specific antigen (PSA)	10^−3^ to 10 ng/mL		[40]
Multilayer MoS_2_	PSA	3.75 nM to 375 fM		[41]
Multilayer MoS_2_	PSA	10^−5^ to 1 ng/mL	100 fg/mL	[42]
Few-layer MoS_2_	PSA	10^−6^ to 100 ng/mL	1 fg/mL	[43]
Multilayer MoS_2_	SARS-CoV-2 spike protein	10^−6^ to 1 ng/mL		[16]
Vertically aligned MoS_2_	PSA	10^−2^ to 10 ng/mL	800 fg/mL	[44]
Multilayer MoS_2_	IgG	10^−3^ to 10^−1^ ng/mL		[45]
Graphene	Bone gla protein	10^−5^ to 10^2^ ng/mL	100 fg/mL (in 0.5% serum)	[46]
Graphene	SARS-CoV-2 Spike S1 antigen	1 fM to 1 μM	10 fM	[47]
Porous graphene	SARS-CoV-2 virus	1 to 10^6^ pg/mL	1 pg/mL (1 ng/mL in human serum)	[48]
Monolayer graphene	SARS-CoV-2 antibody	5 aM to 5 pM	0.39 fg/mL	[49]
Vertically oriented graphene	HIgG	2 to 20 ng/mL	2 ng/mL	[50]
GM	HIgG	10^−4^ to 10^6^ ng/mL	12.4 fg/mL	This work
MG	HIgG	10^−4^ to 10^6^ ng/mL	26.4 fg/mL	This work

## Data Availability

Detailed data can be obtained from the authors.
